# Supramolecular clustering of the cardiac sodium channel Nav1.5 in HEK293F cells, with and without the auxiliary β3‐subunit

**DOI:** 10.1096/fj.201701473RR

**Published:** 2020-01-16

**Authors:** Samantha C. Salvage, Johanna S. Rees, Alexandra McStea, Michael Hirsch, Lin Wang, Christopher J. Tynan, Matthew W. Reed, Jennifer R. Irons, Richard Butler, Andrew J. Thompson, Marisa L. Martin-Fernandez, Christopher L.‐H. Huang, Antony P. Jackson

**Affiliations:** ^1^ Deparment of Biochemistry University of Cambridge Cambridge UK; ^2^ Central Laser Facility Research Complex at Harwell Science and Technology Facilities Council Rutherford Appleton Laboratory Oxford UK; ^3^ Department of Nuclear Physics Research School of Physics and Engineering Australian National University Canberrra ACT Australia; ^4^ Wellcome Trust/Cancer Research UK Gurdon Institute University of Cambridge Cambridge UK; ^5^ Department of Pharmacology University of Cambridge Cambridge UK; ^6^ Department of Physiology, Development and Neuroscience University of Cambridge Cambridge UK

**Keywords:** β3‐subunit, Hodgkin Huxley kinetics, super‐resolution imaging, voltage‐gated sodium channel Nav1.5

## Abstract

Voltage‐gated sodium channels comprise an ion‐selective α‐subunit and one or more associated β‐subunits. The β3‐subunit (encoded by the SCN3B gene) is an important physiological regulator of the heart‐specific sodium channel, Nav1.5. We have previously shown that when expressed alone in HEK293F cells, the full‐length β3‐subunit forms trimers in the plasma membrane. We extend this result with biochemical assays and use the proximity ligation assay (PLA) to identify oligomeric β3‐subunits, not just at the plasma membrane, but throughout the secretory pathway. We then investigate the corresponding clustering properties of the α‐subunit and the effects upon these of the β3‐subunits. The oligomeric status of the Nav1.5 α‐subunit in vivo, with or without the β3‐subunit, has not been previously investigated. Using super‐resolution fluorescence imaging, we show that under conditions typically used in electrophysiological studies, the Nav1.5 α‐subunit assembles on the plasma membrane of HEK293F cells into spatially localized clusters rather than individual and randomly dispersed molecules. Quantitative analysis indicates that the β3‐subunit is not required for this clustering but β3 does significantly change the distribution of cluster sizes and nearest‐neighbor distances between Nav1.5 α‐subunits. However, when assayed by PLA, the β3‐subunit increases the number of PLA‐positive signals generated by anti‐(Nav1.5 α‐subunit) antibodies, mainly at the plasma membrane. Since PLA can be sensitive to the orientation of proteins within a cluster, we suggest that the β3‐subunit introduces a significant change in the relative alignment of individual Nav1.5 α‐subunits, but the clustering itself depends on other factors. We also show that these structural and higher‐order changes induced by the β3‐subunit do not alter the degree of electrophysiological gating cooperativity between Nav1.5 α‐subunits. Our data provide new insights into the role of the β3‐subunit and the supramolecular organization of sodium channels, in an important model cell system that is widely used to study Nav channel behavior.

**Abbreviations**
AFM: atomic force microscopy
BrS: Brugada syndrome
*C*_m_: membrane capacitance
Co‐IP: co‐immunoprecipitation
DDM: n‐dodecyl β‐D‐maltoside
DTT: dithiothreitol
*E*_Na_: Na^+^ reversal potential
EGFP: enhanced green fluorescent protein
ER: endoplasmic reticulum
ERAD: endoplasmic reticulum associated degradation
FBS: fetal bovine serum
FRET: fluorescence resonance emission transfer
*G*: conductance
*G*/*G*_max_: normalized conductance
HBSS: Hanks’ balanced salt solution
HEK: human embryonic kidney cells
*I*: current
Ig: immunoglobulin
LQTS: long QT syndrome
Nav1.5: cardiac voltage‐gated sodium channel
PBS: phosphate‐buffered saline
PEI: polyethylenimine
PFA: paraformaldehyde
PLA: proximity ligation assay
PSF: point spread function
SEM: standard error of the mean
STORM: stochastic optical reconstruction microscopy
TCEP: tris(2‐carboxyethyl) phosphine
TBST: tris buffered saline with Tween20
β3: voltage‐gated sodium channel beta 3 subunit

## 1 INTRODUCTION

The cardiac voltage‐gated sodium channel (Nav1.5) is crucial for the generation and conduction of the cardiac action potential. Nav1.5 is a transmembrane protein that comprises a large pore‐forming, sodium‐selective α‐subunit that contains four homologous domains (DI‐DIV). Each domain consists of six membrane spanning alpha helices (S1‐S6). In each domain, helices S1‐S4 form the voltage‐sensor regions. The S4 helix contains positively charged residues that enable it to move in response to changes in the membrane potential. This movement drives further conformational changes, leading to transient channel opening, quickly followed by channel inactivation.^1^ Movement of the S4 segments of DI‐DIII has been associated with the activation step, whereas S4 of DIV is associated with inactivation, consistent with the m^3^h model of Hodgkin and Huxley.^2^

There are four Nav channel β‐subunit genes (SCN1B‐4B) encoding proteins β1‐β4.^3^ All the β‐subunits—except an alternatively spliced β1b isoform—contain an extracellular immunoglobulin (Ig) domain connected *via* a small flexible neck to a single transmembrane spanning alpha‐helical region and a short intracellular C‐terminal tail. The β‐subunits influence Nav channel activity through effects on the voltage sensitivity of activation and inactivation, the kinetics of channel activation and inactivation, as well as indirect effects such as alterations in the trafficking of channels from the endoplasmic reticulum (ER) to the plasma membrane. However, individual β‐subunit isoforms modify these parameters to different extents, often in a cell‐specific manner.^4^, ^5^, ^6^

Expression of the β3‐subunit is most abundant in the ventricles of the heart^4^ and the importance of β3‐subunit regulation of Nav1.5 is particularly apparent in ventricular arrhythmic syndromes, including Brugada syndrome (BrS) and Long QT syndrome. This has been experimentally modeled in the β3‐knockout (*Scn3b^−/−^*) murine cardiac model which shows a BrS‐like phenotype that includes a reduction of peak sodium current density (*I*_Na_), abnormal ventricular electrophysiological characteristics, alterations in sino‐atrial node (SAN) function and abnormal atrial conduction.^7^, ^8^ Similarly, a number of SCN3B gene mutations have been identified in cases of BrS in which the Nav1.5 protein is apparently normal.^9^

The atomic‐resolution structure of the β3‐subunit Ig domain suggests that it can form trimers when expressed in HEK293F cells. Moreover, images of immuno‐purified channels obtained by atomic force microscopy (AFM) indicate that the β3‐subunit can bind at up to four sites, symmetrically arranged around the Nav1.5 α‐subunit.^10^ This implies a more complex and perhaps variable stoichiometry between these two channel components than has traditionally been assumed. However, it is not yet clear if this complexity can contribute to higher‐order associations between Nav1.5 channels.

Here we use cell‐biological, biochemical, and super‐resolution imaging approaches to further investigate the oligomeric status of the β3‐subunit and its effect on the *in vivo* organization of Nav1.5 α‐subunits. We find that in the HEK293F cell‐line model, commonly used in electrophysiological assays, the Nav1.5 α‐subunit forms oligomeric complexes on the plasma membrane even in the absence of β3. However, the β3‐subunit does modulate the organization of individual Nav1.5 α‐subunits within the clusters while not altering the degree of gating cooperativity between individual α‐subunits. Our work identifies an unexpected property of Nav1.5 channels in a cell system routinely used for electrophysiological studies and raises new questions about the control of Nav channel clustering.

## 2 MATERIALS AND METHODS

### 2.1 Cell culture, DNA constructs, and transfections

Human embryonic kidney (HEK293F) cells and HEK293F cells stably expressing Nav1.5 (HEK293F‐Nav1.5) were maintained in DMEM (DMEM/F‐12 Glutamax, Invitrogen, UK) with 10% FBS (Sigma‐Aldrich, UK) at 37°C and 5% CO_2_. The plasmids pcDNA3‐Nav1.5‐hemagglutinin (HA), pcDNA3 Nav1.5‐green fluorescent protein (GFP), pEnhanced Green Fluorescent Protein (EGFP), pEGFP‐β3, and pFBM β3‐myc have all been previously described.^11^, ^12^, ^13^ Transient transfections were performed using polyethylenimine (PEI, 1 µg/µl) at a PEI/DNA ratio of 3:1.

For whole cell patch clamp electrophysiology, HEK293F‐Nav1.5 cells were plated on 18 mm coverslips in six‐well plates and transiently transfected with either 1 µg of the empty vector pEGFP‐N1 or pEGFP‐β3.

Transient transfections for biochemical experiments were carried out on either HEK293F or HEK293F‐Nav1.5 in 100 mm dishes at 70%‐80% confluency. For co‐immunoprecipitation studies, HEK293F cells were transfected with either 4 µg of β3‐EGFP or β3‐myc alone or co‐transfected with 4 µg each. Proximity ligation assay (PLA) and immunohistochemistry experiments were performed on HEK293F cells transfected with 3 µg each of Nav1.5 HA and Nav1.5 EGFP ± 3 µg of β3‐myc.

For STORM experiments, HEK293F cells were plated in 35 mm glass (no. 1) bottom dishes and transfected at around 70% confluency with Nav1.5 HA (0.5 µg) and either β3‐EGFP (0.5 µg) or EGFP (0.5 µg).

### 2.2 Co‐immunoprecipitation

Forty‐eight hours after transfection, cells were washed 3× in cold phosphate‐buffered saline (PBS, ThermoFisher, UK) then lysed in 1 mL lysis buffer (Tris 50 mM, NaCl 150 mM, 1% Triton x‐100 (v/v)) supplemented with protease inhibitor cocktail (Roche, Sigma‐Aldrich, UK). Lysates were vortexed and mixed with end‐over‐end rotation at 4°C for 30 minutes, centrifuged at 10 000 *g* for 10 minutes at 4°C and the pellet (cell debris) fraction discarded. Lysates were incubated with mouse anti‐myc tag, mouse anti‐GFP tag, or mouse anti‐HA tag with end‐over‐end rotation at 4°C overnight, followed by the addition of Protein G agarose for 4 hours. The samples were spun at 2000 *g* at 4°C for 5 minutes. Pellet (bound) fractions were washed 4× in 1 mL lysis buffer and both these and the supernatants (unbound) were incubated in 4× NuPage loading buffer supplemented with dithiothreitol (DTT) at 85°C for 10 minutes. The bound and unbound fractions were separated on SDS‐PAGE, transferred to nitrocellulose membranes (iBlot transfer system, ThermoFisher Scientific) and Western blots carried out with rabbit polyclonal antibodies; anti‐EGFP (GeneTex, Insight Biotech, UK) and anti‐myc (Santa Cruz, Insight Biotech, UK) were used to detect β3‐EGFP and β3‐myc, respectively.

### 2.3 Cross‐linking experiments

HEK293F cells transfected with 4 µg of either β3‐EGFP or β3‐myc were washed 3× in Hanks' balanced salt solution (HBSS, ThermoFisher, UK) and lysed in 1 mL of HBSS lysis buffer (1% Triton x‐100, 0.2% sodium dodecyl sulfate (SDS), 0.5% sodium deoxycholate) with protease inhibitors (Complete Protease inhibitor cocktail, Roche). The lysates were split into two equal fractions: a control had nothing added, and to the other, 5 mM bis(sulfosuccinimidyl) suberate (BS3; an amine‐to‐amine cross‐linker) was added and incubated at 4°C for 1 hour with end‐over‐end rotation. The reaction was quenched with 74 mM glycine and clarified at 10 000 *g* for 10 minutes at 4°C. Samples were then subject to SDS‐PAGE and Western blot as previously described.

### 2.4 Super‐resolution STORM imaging

HEK293F‐Nav1.5 cells co‐transfected with Nav1.5‐HA and β3‐EGFP or EGFP were washed 3× in cold PBS and then fixed with 4% paraformaldehyde (PFA) for 10‐15 minutes. PFA was rinsed off with PBS, the cells were permeabilized in 0.1% (v/v) Triton x‐100 in PBS for 10 minutes followed by blocking with 1% BSA for 1 hour at room temperature. Primary antibody (mouse monoclonal anti‐HA, sc‐7392, Santa Cruz) was incubated at 4°C overnight, followed by, 4× 10‐15 minutes washes in PBST (PBS supplemented with 0.1% Tween20). Secondary antibody (goat anti‐mouse IgG Alexa647, Invitrogen, ThermoFisher, UK) was incubated for 1 hour at room temperature followed by 4× 10‐15 minutes washes in PBST and stored in PBS at 4°C until ready for STORM.

The super‐resolution STORM images were taken on a Zeiss Elyra PS.1 system. The fluorophore Alexa Fluor 647 was photo‐switched using a 642 nm excitation laser, with 100 mM dithiothreitol in PBS as the switching buffer. The power density of the 642 nm illumination on the sample plane was about 5.5 kW/cm^2^. A 100× NA 1.46 oil immersion objective lens (Zeiss alpha Plan‐Apochromat) with a two‐color notch filter (561/642 nm) was used as dichroic mirror in the imaging. The final fluorescent images were projected on an Andor iXon 897 EMCCD camera.

Super‐resolution STORM images were reconstructed and rendered in ZEISS ZEN software. The PALM function in ZEN allows filters to be set for peak finding and choosing a fit model for the localization calculation. In STORM image processing, 9 pixels of peak mask size were applied according to pixel resolution and PSF size in the data sets. The peak intensity to noise filter was set to 6 as this was efficient to identify peaks from background. The fit model was a two‐dimensional Gaussian fit, and only single emitters from fluorophores were taken into account, whereas all multiple emitters were discarded. Following the localization, the displacements of molecules from drifts in the reconstructed images were corrected for using feature detection and cross correlation.

The clustering analysis was conducted as described in Roberts et al,^14^ data analysis section A “Analysis using Bayesian Cluster Algorithm.” We selected 12 regions of interest (ROIs), with a total of 199 589 observations for cells expressing Nav1.5 alone and 16 ROIs with a total of 272 678 observations for cells co‐expressing Nav1.5 and β3‐subunit. The algorithm reports for each cluster the cluster radius and the number of molecules in the cluster. The summaries include the data of all clusters found in the ROIs. The nearest‐neighbor analysis employs a standard k‐d tree. The nearest neighbor for all observations in the ROIs was calculated as described.^14^ The nearest neighbor may have been outside the ROI where the observation was located near the edge of the ROI. Comparisons of radii and nearest‐neighbor distributions for clusters with Nav1.5 alone and clusters with Nav1.5 and β3‐subunit were tested for whether these distributions were identical using the two‐sample Kolmogorov‐Smirnov (KS) test, which compares the samples from the two conditions without the need for a reference distribution.^15^

### 2.5 Proximity ligation assay

Twenty‐four hours after transfection, cells were transferred to poly‐l‐lysine‐coated glass coverslips and left for a further 24 hours. Cells were then washed 3× in cold phosphate‐buffered saline (PBS, ThermoFisher, UK) and fixed with 4% paraformaldehyde (PFA) for 10‐15 minutes. PFA was rinsed off with PBS, the cells were permeabilized in 0.1% (v/v) Triton x‐100 in PBS for 10 minutes followed by blocking with 1 % BSA for 1 hour at room temperature. Primary antibodies were incubated at 4°C overnight and the procedure carried out according to the manufacturer's instructions (Duolink, Sigma). Briefly, cells were washed, then incubated with PLA probes (specific to the species of the primary antibodies) at 37°C in a humidity chamber. This was followed by a 30 minute ligation step at 37°C, and an amplification step of 100 minutes at 37°C with intervening washes. Coverslips were mounted on glass slides with the supplied DAPI mounting media, sealed with clear nail polish, and viewed on an Olympus FV1000 confocal microscope. DAPI, GFP, and AlexaFluor594 were observed. The 488 and 559 nm laser lines were used in a sequential manner to prevent bleed through of signals. A custom Python script for Fiji^16^ was used to enable automated analysis of the PLA data. The script maps the volumes of DAPI and Nav1.5‐GFP signals using a 3D Gaussian blur (sigma = DAPI; 0.5 and EGFP; 0.25) to reduce the effects of noise and the Triangle thresholding algorithm to generate binary masks. PLA signal dots, indicative of protein proximity, were mapped by subtracting the 3 × 3 × 1 3D maximum filtered signal from the original, then detecting local maxima with a noise tolerance of 15. These detected points were used to measure the values of the exact signed Euclidean distance transform of the DAPI mask as well as the signal intensity values in the Nav1.5‐GFP and PLA channels. These measurements allowed us to calculate the PLA dot count per Nav1.5‐GFP‐positive cell. In addition, PLA signal intensity was determined using the online freely available software “BlobFinder”.^17^

### 2.6 Whole cell patch clamp

Na^+^ currents (*I*_Na_) were recorded from HEK293F cells stably expressing Nav1.5 and transiently transfected with either a vector containing only EGFP (Nav1.5 + EGFP) or β3‐EGFP (Nav1.5 + β3‐EGFP). Successfully transfected cells were identified by EGFP fluorescence on an Olympus IX71 inverted microscope. Experiments were carried out at room temperature (22‐23°C) in the whole cell configuration with an Axopatch 200B amplifier (Axon instruments, California, US), a Digidata 1322A digitizer (Axon instruments, California, US), and the Strathclyde Electrophysiology Software Package (WinWCP, Department of Physiology and Pharmacology, University of Strathclyde). The extracellular bath solution contained (in mM) the following: NaCl 60, KCl 2, CaCl_2_ 1.5, glucose 10, MgCl_2_ 1, CsCl_2_ 90, HEPES 10, pH 7.39 ± 0.02 with NaOH. 1.5‐2.5 MΩ patch pipettes were produced from borosilicate glass capillaries (Harvard Apparatus Ltd, UK) using a horizontal puller (P‐87 Sutter Instruments, CA, USA) and filled with intracellular saline, comprising (in mM) the following: NaCl 35, CsF 105, EGTA 10, HEPES 10, pH 7.39 ± 0.02 with CsOH. Signals were low‐pass Bessel filtered at a frequency of 5 kHz and sampled at 125 kHz. Series resistance compensation was performed to 75%‐80% and leak currents were subtracted using a P/4 protocol. The liquid junction potential (2 mV) was not corrected for. Data from cells with a current amplitude larger than 8 nA, or with a clear loss of voltage control as demonstrated by poor *I*/V relationships were removed.

### 2.7 Voltage protocols

Voltage protocols used a holding voltage of −120 mV. The activation protocol consisted of a 50 ms depolarizing test pulse ranging from −90 mV to +35 mV, in 5 mV increments. The resulting current traces were normalized against the whole cell capacitance (*C*_m_) and the *I/V* relationship plotted from peak current at each test voltage *V*. Values of Na^+^ conductance (*G*_Na_), for families of traces at each test voltage, were determined from the equation:

(1) $$G_{\text{Na}}=I_{\text{Na}}/\left(V-E_{\text{Na}}\right)$$

where *I*_Na_ is the Na^+^ current and *E*_Na_ is the Na^+^ reversal potential. Peak *G*_Na_ was plotted as a function of voltage to produce activation curves.

Steady‐state inactivation was assessed with 100 ms conditioning pre‐pulses ranging from −140 to −50 mV in 5 mV increments followed immediately by a 50 ms depolarizing test pulse to −40 mV. *I*_Na_ was normalized to the maximum elicited current and plotted against the conditioning voltage to yield inactivation curves. Both curves were fitted to the following Boltzmann function:

(2) $$G/G_{\max}=1/(1+\exp\left(\left(V-V_{1/2}\right)/k)\right)$$

where *G*/*G*_max_ is the normalized conductance or current, *V*_½_ is the voltage of half‐maximal activation or inactivation, *k* is the slope factor, and *V* is the test voltage or conditioning voltage.

Recovery from inactivation was examined using a double pulse protocol that delivered two identical depolarizing pulses to −40 mV (P1 and P2) of 50 ms duration. The time interval between P1 and P2 was incremented by 3 ms with each successive sweep up to a maximum interval of 72 ms. Peak currents from P2 were normalized to those obtained in response to the conditioning P1 step and plotted against the time intervals. These plots were fitted with a mono‐exponential function as follows:

(3) $$I_{\text{P2}}/I_{\text{P1}}=1-\exp\left(-t/\tau \right)$$

where *t* is the time and τ is the time constant of recovery from inactivation.

### 2.8 Analysis of Na^+^ current kinetics

Families of *G*_Na_, in response to each voltage step or conditioning voltage, were then fitted to the standard solution for the time course of *G*_Na_ employing the established Hodgkin‐Huxley *m* ^3^ *h* formulation:

(4) $$G_{\text{Na}}=\bar{G_{\text{Na}}}{\left(m_{\infty }-\left(m_{\infty }-m_{0}\right)e^{-t/\tau_{m}}\right)}^{3}\left(h_{\infty }-\left(h_{\infty }-h_{0}\right)e^{-t/\tau_{h}}\right)+\text{offset}$$

where the maximum steady‐state conductance term $\bar{G_{\text{Na}}}$ was derived from the voltage step that yielded the maximum peak *I*_Na_ in the patch studied. The terms *m* and *h* represent the Hodgkin‐Huxley activation and inactivation variables. These have time constants τ*_m_* and τ*_h_*, respectively, describing the decay in their respective variables in response to either the activating or inactivating voltage step imposed at time = 0, to the steady state, *t* = ∞, following the voltage step. The offset term in the equation allowed for the finite membrane conductance. The gating variables, *m*_0_ and *m*_∞_, were obtained from the cube root of the previously determined *G*/*G*_max_ values from the activation function (Equation 2) whose values of *V_½_* and *k* were derived at each voltage preceding and immediately following the voltage step. Similarly, *h*_0_ and *h*_∞_ were obtained directly from the *G*/*G*_max_ values in the steady‐state inactivation function for the voltage preceding and immediately following the voltage step. These fits allowed for the derivation of the activation (τ_m_) and inactivation (τ_m_) decay constants.

The time course of current decay was determined using the following double exponential fit:

(5) $$y=-A_{1}\exp\left(-t/\tau_{1}\right)-A_{2}\exp\left(-t/\tau_{2}\right)$$

where *A*_1_ and *A*_2_ are the magnitudes of the fast and slow currents, respectively, and τ_1_ and τ_2_ are the corresponding time constants. Peak currents were measured using WinWCP v 5.0.9 and analyzed in Prism v.7. For *m* ^3^ *h* analysis and time constants of inactivation, raw data were exported and analyzed through a custom Python script.

## 3 RESULTS

### 3.1 Oligomerization of the β3‐subunit when expressed in HEK293F cells

**Figure 1:**
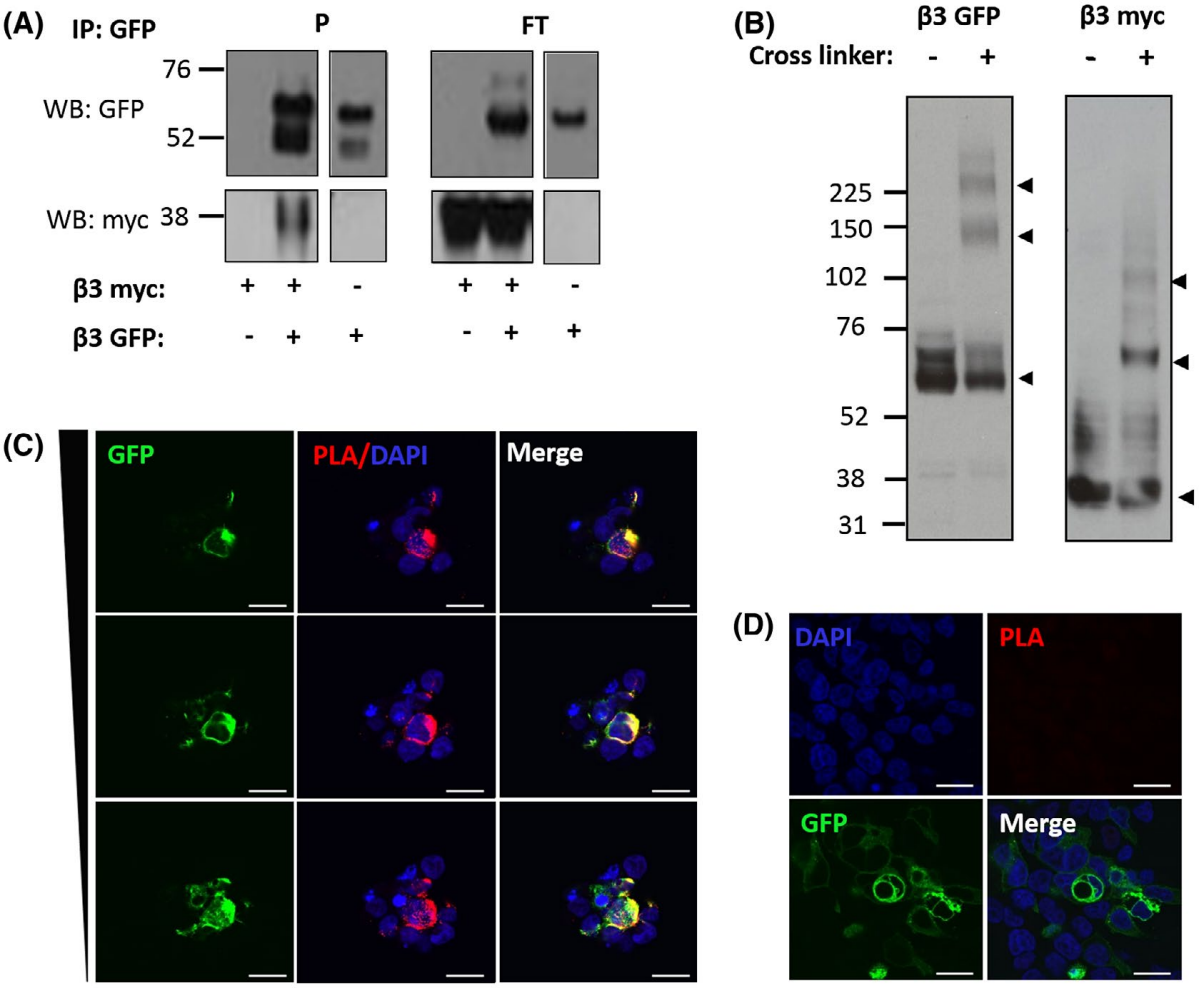
Oligomerization of β3. A, Co‐IP of overexpressed β3‐EGFP and β3‐myc from singly or co‐transfected HEK293F cell lysates. β3‐myc only appeared in the bound fraction when co‐expressed with β3‐EGFP. P = “pellet,” that is, bound fraction; FT = “flow through,” that is, non‐bound fraction. B, Cross‐linking of singly expressed β3‐EGFP and β3‐myc subunits. In the absence of the cross‐linking agent (BS3), only bands consistent with the monomer (lower arrow‐head), ~56‐60 and ~36‐38 kDa for β3‐EGFP and β3‐myc, respectively, and glycosylation states were observed. Chemical cross‐linking resulted in multiple bands, consistent with the monomeric, dimeric (~120 and ~72 kDa, middle arrowhead) and trimeric (~180 and ~110 kDa, upper arrowhead) forms, and glycosylation states. C, Proximity ligation assay (PLA) of β3‐myc and β3‐EGFP. Oligomerization of β3 in situ is demonstrated by the amplification of PLA signals, seen throughout the cell and plasma membrane through a series of z‐stacks (left‐hand panel: top to bottom at 1.8 µm intervals), when β3‐myc is co‐expressed with β3‐EGFP. D, Expression of β3‐EGFP alone (right‐hand panels) did not produce the characteristic punctate red signal typical of PLA tagged proteins within 40 nm of each other. Scale bar = 20 µm. Distance through the z stack is indicated by the tapering bar on the left‐hand side of panel C

We have previously shown that the β3‐subunit, expressed in HEK293F cells can form trimers on the plasma membrane surface.^10^ However, the stability and extent of this interaction is not clear. Neither is it clear whether the β3‐subunit oligomerization occurs only on the plasma membrane. To investigate these questions, we performed co‐immunoprecipitation on lysates from HEK293F cells co‐transfected with two differently tagged β3 constructs: β3‐EGFP and β3‐myc. As a control, single transfection of the β3‐myc construct was performed. The smaller size of myc relative to EGFP results in a mobility shift which allows for the discrimination of the two β3‐subunits on a Western blot; β3‐myc migrates further than β3‐EGFP (30‐35 kDa shift). The β3‐EGFP subunit was immunoprecipitated with a mouse anti‐GFP antibody/protein G agarose mixture and found in the bound fraction in all cell lysates in which it was transfected (Figure 1A). This mixture was saturated and thus β3‐EGFP was also found in the unbound supernatant fraction. Crucially, co‐immunoprecipitation of β3‐myc only occurred in the presence of β3‐EGFP; when β3‐myc was transfected alone, it was only found in the unbound, supernatant fraction (Figure 1A).

The cell lysates from cells separately transfected with β3‐myc and β3‐EGFP were cross‐linked with BS3 and examined by Western blotting (Figure 1B). Cross‐linking of β3‐myc produced large distinct bands at approximately 35, 70, and 105 kDa, whereas cross‐linking of β3‐EGFP resulted in bands at approximately 60, 120, and 180 kDa; in both cases indicative of monomers, dimers, and trimmers, respectively. There was additional, less intense, smearing traveling upwards from the monomeric bands in both samples, and to a lesser degree in both the dimeric and trimeric bands, which are likely post‐translational modifications, such as glycosylation. The different migratory patterns between the two tagged β3‐subunits can be attributed to the differing sizes of the myc (1‐2 kDa) and EGFP (30‐32 kDa) tags. Thus, the oligomeric interaction between β3‐subunits is sufficiently stable to survive cell lysis and the prolonged washing steps inherent in immunoprecipitation experiments. Furthermore, under these conditions, the β3‐subunits are capable of forming dimers and trimers, rather than aggregates of indeterminate stoichiometry.

To assess the cellular compartments where β3‐subunit oligomerization occurs in situ*,* we used the PLA. This method employs two antibodies from different species targeting different epitope tags on co‐expressed β3‐subunits. Secondary antibodies raised against each primary antibody and conjugated to a matched pair of short single‐stranded oligonucleotides are added. If the two respective targets are within about 40 nm, the oligonucleotide probes will hybridize with a “bridging oligonucleotide” to form a continuous circular DNA structure, which can be amplified by DNA polymerase and detected by a fluorescently labeled oligonucleotide.^18^ Co‐transfection of β3‐EGFP and β3‐myc, each tagged with one of the PLA probes post‐fixation, produced characteristic punctate staining in the red (597 nm) channel, indicating that co‐transfected β3‐EGFP and β3‐myc resided within 40 nm of each other (Figure 1C). Labeling was heavy and not just restricted to the plasma membrane, but also occurred internally, especially in large perinuclear regions consistent with ER and other compartments within the secretory pathway. No discernible PLA signal was observed when either β3‐EGFP or of β3‐myc was expressed alone, indicating that the PLA probes showed no non‐specific binding (Figure 1C). Thus, the β3‐subunits have a natural tendency to oligomerize when expressed in HEK293F cells, and this occurs at an early part of the secretory pathway.

### 3.2 Formation of Nav1.5 complexes in situ

**Figure 2:**
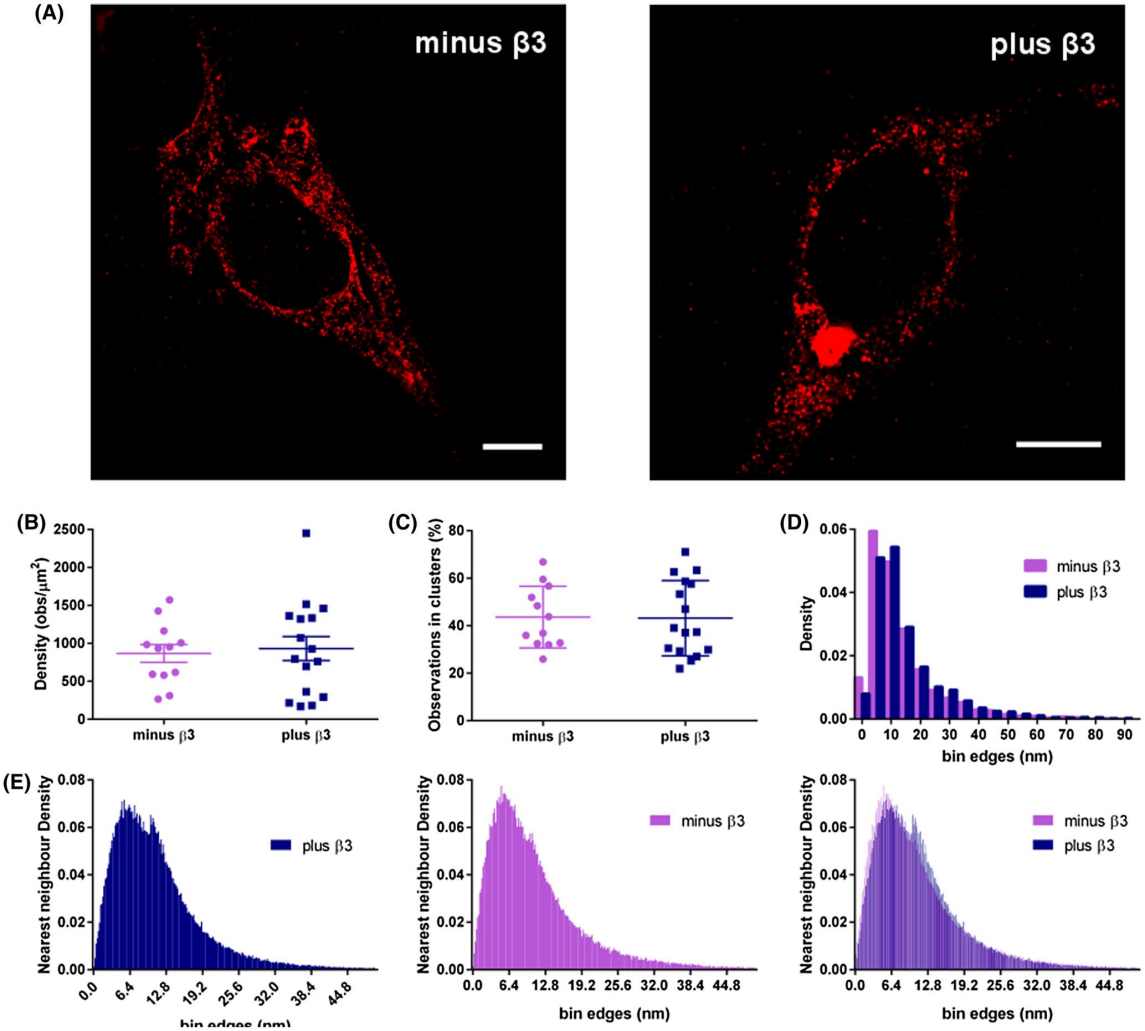
Super‐resolution STORM imaging and cluster and nearest‐neighbor analysis of Nav1.5 α‐subunits with and without β3‐subunit. A, Representative reconstructed images of fluorophore AF‐647 blinking in HEK293F cells co‐transfected with Nav1.5‐HA/EGFP and Nav1.5‐HA/β3‐EGFP. Bars = 5 µm B, The percentage of total blinking events that were observed in the same area (cluster) as other blinking events (ie, reflecting co‐localized Nav1.5 channels) in the presence and absence of β3. C, The density of blinking events observed per unit area (µm^2^) for cells expressing Nav1.5‐HA, plus and minus β3. D, Normalized cluster radii distribution density for cells expressing Nav1.5‐HA plus and minus β3. E, Normalized nearest‐neighbor distance density between Nav1.5‐HA molecules for cells expressing Nav1.5‐HA, plus and minus β3, both individually and superimposed. In D, differences between the histogram distributions with and without β3 were tested using the two‐sample Kolmogorov‐Smirnov test, giving a *P* value of 4 × 10^−9^. In E, differences between the histogram distributions with and without β3 were tested using the two‐sample Kolmogorov‐Smirnov test, giving a *P* value of ~0

To examine the oligomeric state of Nav1.5, we transiently expressed HA‐epitope tagged Nav1.5 α‐subunits in HEK293F cells, with or without EGFP‐tagged β3‐subunit and examined their distribution on the plasma membrane using super‐resolution STORM imaging. Transfected cells were fixed and permeabilized and labeled with a monoclonal antibody against HA epitope, followed by Alexa Fluor 647‐labeled second antibody as described in Materials and Methods. Typical labeling at the plasma membrane is shown in Figure 2A. Surprisingly, Nav 1.5 α‐subunits alone were not randomly dispersed on the plasma membrane but about 40% of the α‐subunits assembled into larger multi‐molecular clusters. The β3‐subunit did not affect the proportion of oligomerized Nav1.5 α‐subunits nor did it affect the density of clusters on the plasma membrane (Figure 2B,C). However, the β3‐subunit did increase the proportion of larger‐radii clusters (Figure 2D). This difference was highly statistically significant (two‐sample KS test, *P* = 4 × 10^−9^). For the case of Nav1.5 α‐subunits expressed alone, the distribution of nearest‐neighbor distances between α‐subunits within clusters followed a positively skewed distribution, with a modal value ~6 nm. This pattern was still present in the nearest‐neighbor distribution measured in the presence of the β3‐subunit. However, now an additional peak with a modal value ~12 nm was also prominent (Figure 2E). This difference was again highly significant (two‐sample KS test, *P* ~ 0).

**Figure 3:**
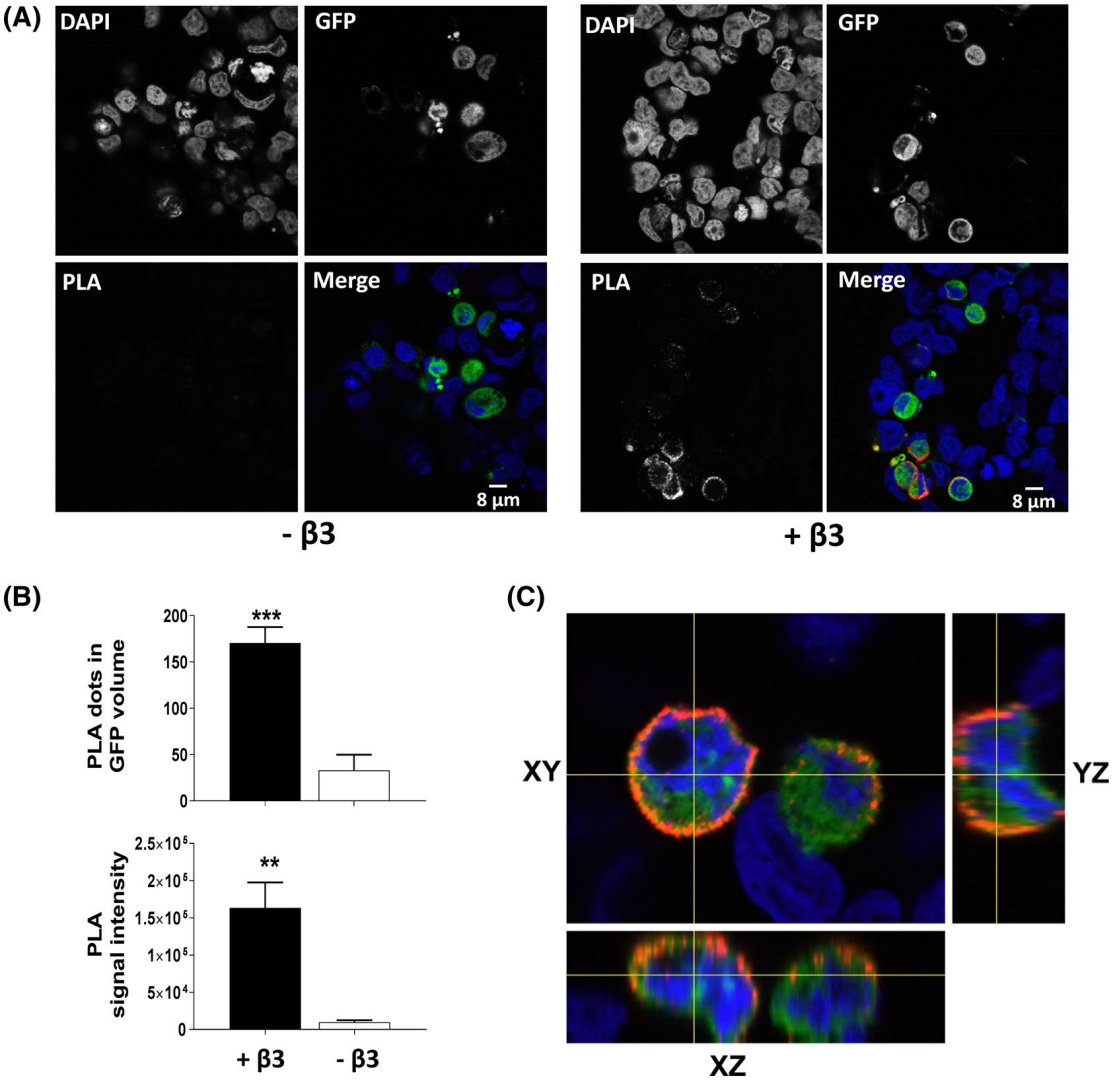
Enhanced oligomerization of Nav1.5 α‐subunit by β3. A, Representative PLA images of Nav1.5 EGFP and Nav1.5 HA in the absence (left‐hand panels) and presence (right‐hand panels) of β3‐myc. Although not visible to the naked eye, a number of PLA signals can be detected by ImageJ and blobfinder in the absence of β3 and is recorded in the histograms. B, Quantification of PLA “dots” within the EGFP volume and PLA signal intensity (mean ± SEM). C, Orthogonal views from sequential images of the PLA signal (red) through the z‐stack in cells expressing Nav1.5 EGFP, Nav1.5 HA, and β3‐myc, highlighting a predominant, but not exclusive, localization of PLA signal at the plasma membrane. To compare the expression levels of Nav1.5 EGFP and Nav1.5 HA in these cells, see Supplementary Figure S1

For comparison, we also performed a PLA experiment to monitor α‐subunit clustering. Since the efficiency of PLA can be very sensitive, both to the relative positions and relative accessibility of the two separate epitopes on separate proteins,^18^ the method may provide additional and complementary information on protein organization within clusters. We used two differently tagged Nav1.5 α‐subunits: Nav1.5‐HA and Nav1.5‐GFP, co‐transfected with or without β3‐myc (Figure 3A). Although β3 was not required to generate PLA signals, it did increase the number of PLA dots detected (Figure 3A,B). Interestingly, this PLA signal occurred predominantly at the plasma membrane, as observed in z‐stack images (Figure 3C). Immunostaining of Nav1.5‐HA was also carried out to confirm that co‐transfection of the β3‐myc did not alter the expression of Nav1.5‐HA and Nav1.5‐GFP. Here, the level of HA‐ and GFP‐tagged α‐subunits were similar in cells with or without β3 (Supplementary Figure S1). Taken together, this indicates that the β3‐subunit is not required for Nav1.5 α‐subunit clustering *per se* but does significantly influence the geometry and/or the relative orientation of channels within clusters.

### 3.3 Steady‐state activation of the Na^+^ channel is unaffected by its co‐expression with the β3‐subunit

**Figure 4:**
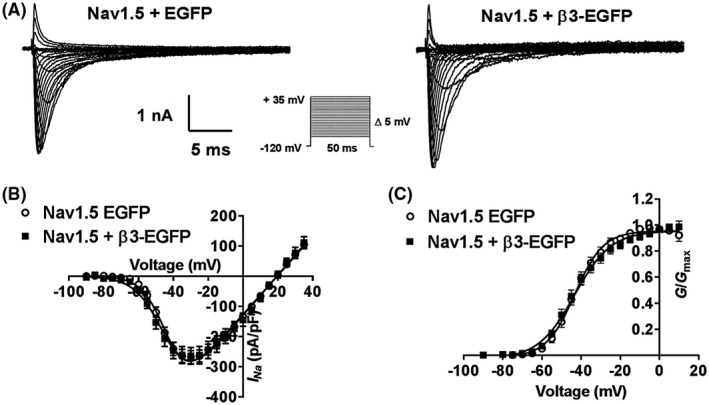
Steady‐state activation in HEK293F‐Nav1.5 cells with and without β3 co‐expression. A, Representative whole cell Na^+^ currents of HEK295F‐Nav1.5 cells transfected with EGFP or β3‐EGFP in response to depolarizing steps (50 ms duration) from a holding potential of −120 mV to test pulses between −90 and +35 mV in 5 mV increments. Cell capacitances of these patches were 10.4 pF and 13.1 pF for Nav1.5 + EGFP and Nav1.5 + B3EGFP, respectively. B, *I/V* curves with currents normalized to cell capacitance for EGFP (open circles) and β3‐EGFP (closed squares) show no effect of β3. C, Channel conductance as a function of voltage fit with a Boltzmann function (Equation 2), showing no shift in *V*_½_ or *k* with β3. Means ± SEM, n = 8 and 10 for Nav1.5 + EGFP and Nav1.5 β3‐EGFP, respectively

Typical examples of whole cell Na^+^ currents (*I*_Na_) from stably expressing HEK293F‐Nav1.5 cells transiently transfected with an empty EGFP vector (Nav1.5 + EGFP) or EGFP‐tagged β3‐subunit (Nav1.5 + β3‐EGFP) in response to an incremental step depolarization are illustrated in Figure 4A. The co‐expression of the β3‐subunit with Nav1.5 did not alter the amplitude of peak current (*I*_Na(max)_; Nav1.5 + EGFP, 263.64 ± 27.70 pA/pF, Nav1.5 + β3‐EGFP, 268.00 ± 21.89 pA/pF, *P* > .05). The current‐voltage relationships yielded *E*_Na_ values in the absence and presence of the β3‐subunit of 19.45 ± 0.98 mV and 19.58 ± 0.68 mV, respectively (Figure 4B)*.* Both of these values are consistent with the value predicted from the Nernst potential (17.46 mV) corresponding to the known extracellular (~70 mM) and intracellular (35 mM) Na^+^ concentrations. Activation curves were normalized to their maximum peak values (giving *G*/*G*_max_) and divided by the driving force (*V*‐*E*_Na_) and yielded similar voltage dependences (*V*_½_; Nav1.5 + EGFP = −42.62 ± 1.81 mV, Nav1.5 + β3‐EGFP = −42.61 ± 1.67 mV, means ± SEMs; *P* > .05), and slope factor (k; Nav1.5 + EGFP = 7.28 ± 0.61 mV, Nav1.5 + β3‐EGFP = 9.42 ± 0.94 mV, *P* > .05; Figure 4B,C). Similarly, the maximal channel conductances (*G*_max_) were similar (*P* > .05, Figure 4C) for Nav1.5 alone (6.47 ± 0.21 mS/cm^2^) and β3‐EGFP (6.69 ± 0.32 mS/cm^2^).

### 3.4 Co‐expression of the β3‐subunit induces a depolarizing shift in the steady‐state voltage dependence of Na^+^ channel inactivation

**Figure 5:**
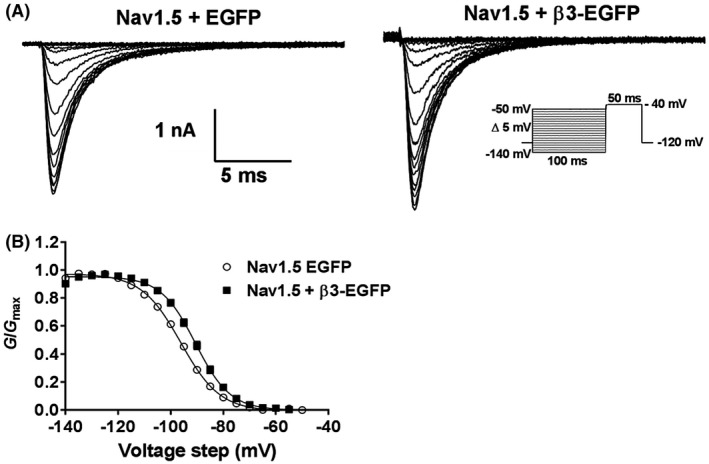
Steady‐state inactivation in HEK293F‐Nav1.5 cells with and without β3 co‐expression. A, Representative whole cell Na^+^ currents of HEK293F‐Nav1.5 cells transfected with EGFP or β3‐EGFP in response to a test pulse of −40 mV (50 ms duration) immediately subsequent to a pre‐pulse step (100 ms duration) to voltages ranging from −140 to −50 mV in 5 mV increments. Cell capacitances of these patches were 10.4 pF and 13.1 pF for Nav1.5 + EGFP and Nav1.5 + β3‐EGFP, respectively. B, Inactivation curves reflecting channel availability (*G*/*G*_max_) as a function of the pre‐pulse voltage step, means ± SEM, n = 27 and 20 for Nav1.5‐EGFP and Nav1.5 + β3‐EGFP, respectively. Solid lines are the fits to a Boltzmann function (Equation 2) highlighting a depolarizing shift in the voltage dependence of inactivation with the presence of β3

Typical inactivation traces from Nav1.5 + EGFP and Nav1.5 + β3‐EGFP are shown in Figure 5A. Co‐expression of the β3‐subunit resulted in a 5.5 mV depolarizing shift of steady‐state inactivation (*V*_½_; Nav1.5 + EGFP = −96.14 ± 0.72 mV; Nav1.5 + β3‐EGFP = −90.64 ± 1.02; *P* < .001; Figure 5B). This depolarizing shift was observed in the absence of any significant variation in k (Nav1.5 + EGFP = −7.17 ± 0.20; Nav1.5 + β3‐EGFP = −6.47 ± 0.32 mV).

### 3.5 Co‐expression of the β3‐subunit does not alter the kinetics of Na^+^ channel activation and inactivation

**Figure 6:**
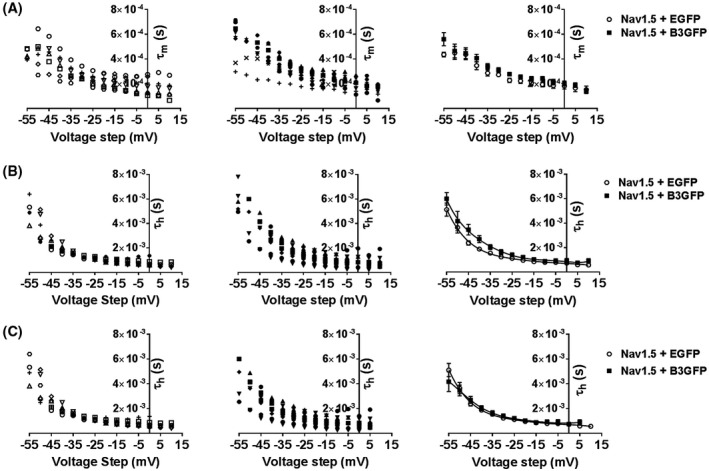
Hodgkin‐Huxley kinetics; time constants of the activation, m, and inactivation, h, gates, from individual experiments with (left panels) and without β3 co‐expression (center panels), and comparison of their mean (+/‐ SEM) results (right panels). A, Time constants of activation (τ_m_). B,C, Time constants of inactivation (τ_h_). B, before and C, following correction of the τ_h_ of the β3 containing HEK293F‐Nav1.5 cells for the observed shift (5.5 mV) in steady‐state inactivation

**Figure 7:**
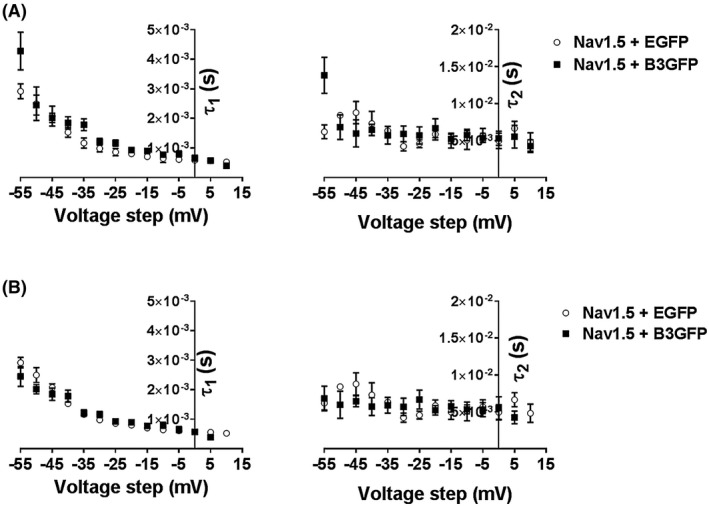
Time constants of inactivation with (open symbols) and without β3 co‐expression (filled symbols). Current decays from the steady‐state activation protocol fitted with a double exponential to give both the fast (τ_1_) (left panels) and slow (τ_2_) time constants of inactivation (right panels) in the presence and absence of the β3‐subunit. A, τ_1_ plotted as a function of test voltage. This demonstrates voltage dependences that are similar whether the β3‐subunit is present or absent (left panel). In contrast τ_2_ is relatively voltage insensitive whether or not β3 is present (right panel). B, Voltage dependences of τ_1_ and τ_2_ following shifting the results obtained in the presence of β3 by −5 mV to allow for the associated depolarizing shift in the steady‐state inactivation

The time constants for activation (*m*) and inactivation (*h*) were determined using the standard Hodgkin Huxley $m^{3}h$ model (Equation 4; Figure 6). The time constants ($\tau_{m}$) for *m* were indistinguishable between Nav1.5 + EGFP and Nav1.5 + β3‐EGFP **(**Figure 6A). Time constants for the inactivation parameter ($\tau_{h}$) appeared to show subtle shifts in the presence of the β3‐subunit (Figure 6B), but when the voltage dependences were corrected for the 5.5 mV shift previously observed in the steady‐state inactivation curves, these time constants were comparable (Figure 6C). However, some reports have described the inactivation process as a sum of two components; a rapid and slow decay.^6^, ^12^ Thus, we further assessed the decay of the Na^+^ current with a double exponential function to find evidence for separate fast (τ_1_) and slow (τ_2_) time constants (Figure 7A), and then similarly corrected for the relative shifts of steady‐state inactivation (Figure 7B). A double exponential fitted the inactivation process better than a single exponential with a fast component that demonstrated a voltage dependence and a slow component that was relatively voltage independent. Nevertheless, neither voltage dependences of such rate constants were altered by the β3‐subunit.

### 3.6 The β3‐subunit accelerates the recovery of the Na^+^ channel from inactivation

**Figure 8:**
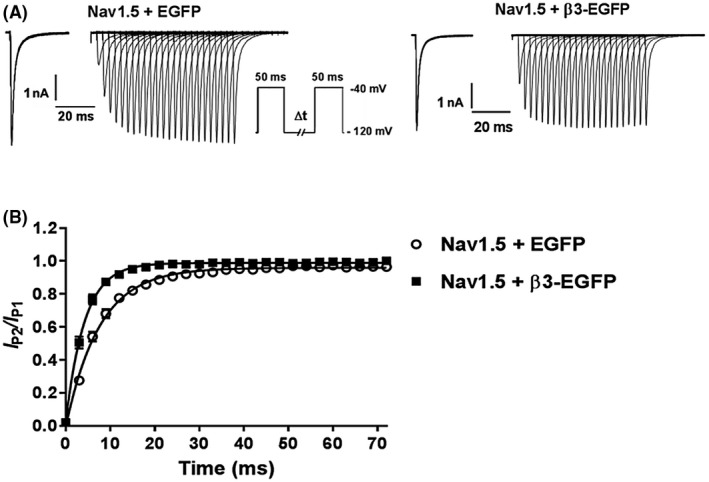
Recovery from inactivation with and without β3 co‐expression. A, Typical whole cell Na^+^ currents of HEK293F‐Nav1.5 cells transfected with EGFP or β3‐EGFP in response to voltage pulse (−40 mV, 50 ms) at varying time intervals (0‐72 ms, in 3 ms increments). B, Peak currents of the test pulse normalized to the conditioning pulse (*I*_P2_/*I*_P1_) plotted as a function of recovery time. Solid lines are mono‐exponential fits highlighting an accelerated recovery time in the β3‐EGFP containing cells. n = 7, Nav1.5 + EGFP; n = 10, Nav1.5 + β3‐EGFP

Typical *I*_Na_ traces from Nav1.5 + EGFP and Nav1.5 + β3‐EGFP in response to a double pulse protocol are shown in Figure 8A. Peak currents of P2 normalized to peak currents of P1 were plotted as a function of the time interval and fitted to a mono‐exponential function. In the presence of the β3‐subunit, there was a significantly accelerated time course for recovery (Figure 8B). The time taken to reach recovery of 50% of the Na^+^ channels was reduced to 2.97 ± 0.82 ms with the β3‐subunit when compared to 5.29 ± 0.92 ms without the β3‐subunit. Consistent with this, the time constant (τ) of recovery was 7.64 ± 1.32 ms for Nav1.5 + EGFP and 4.28 ± 1.19 ms for Nav1.5 + β3‐EGFP.

## 4 DISCUSSION

The atomic‐resolution structure of the β3‐subunit Ig domain indicates that it can trimerize and the isolated Ig domain has been shown to associate in solution with low affinity.^3^, ^10^, ^19^ Super‐resolution imaging has also identified full‐length β3‐subunits assembled as trimers on the plasma membrane of HEK293F cells.^10^ In view of the striking nature of this interaction, and its potential functional implications, we further explored the self‐association of the full‐length β3‐subunit *in situ* as well as its potential to influence Nav channel assembly and function.

Here we show by co‐immunoprecipitation that the oligomeric interaction between β3‐subunits, expressed in the absence of α‐subunit, is sufficiently stable to survive immunoprecipitation. Cross‐linking of these samples is consistent with an existence of β3‐subunit dimers and trimers rather than aggregated protein (Figure 1A,B). PLA analysis provides further evidence that full‐length β3‐subunits, transfected into HEK293F cells can oligomerize. This occurs both at the plasma membrane and within the secretory pathway. Thus, the self‐association begins shortly after biosynthesis (Figure 1C). The trimer interface on the β3‐Ig domain contains an extended hydrophobic patch at its N‐terminal region, which is held exposed on the protein surface by an intra‐subunit disulfide bond.^10^ Proteins within the ER that contain exposed hydrophobic regions are strong candidates for removal and destruction by the ERAD pathway.^20^ Early trimer formation within the ER would bury the hydrophobic N‐terminal region of β3 and facilitate its export and traffic to the surface. *Cis*‐interactions have also been characterized for the Nav channel β4‐subunit, independently of its binding to Nav channels. In this case, the cis‐interactions between β4‐subunits facilitate trans‐cell adhesion behavior.^20^, ^21^, ^22^ The β3‐subunit has also been shown to promote *trans*‐cell adhesion, at least under conditions of high expression.^19^ Hence, the ability of β3‐subunits to homo‐trimerize may reflect a more general tendency of Nav channel β‐subunits to self‐assemble, at least under some *in vivo* conditions—for example, in the absence of Nav α‐subunits.^23^

On neuronal cells and cardiomyocytes, Nav channels are known to be restricted into local micro‐domain regions of the plasma membrane.^24^ Furthermore, it has been reported that that these channels may functionally interact under pathological conditions.^24^, ^25^ However, in these cases, Nav channel clustering is assumed to reflect heterogeneous and multi‐valent molecular interactions between Nav channels themselves and many different cell‐specific proteins such as gap‐junction proteins, cell‐adhesion molecules, cytoskeletal components, and other ion channels.^26^

Previous evidence suggests that heterologously expressed Nav1.5 α‐subunits can assemble as dimers.^27^, ^28^, ^29^, ^30^, ^31^ The epitope recognized by the antibody used in our STORM experiments is located at the cytoplasmic C‐terminus of the Nav1.5 α‐subunit. By comparison with other Nav channels whose near atomic‐resolution structure has been resolved, it is likely that this epitope lie close to or underneath the central pore of the channel, albeit with some flexibility.^32^ Observed from above the membrane, a single α‐subunit is an approximate square of length ~8 nm.^33^ Thus, given the uncertainties generated by antibody size and orientation, the single 6 nm modal peak in the nearest‐neighbor distribution and the <10 nm modal peak in the cluster radii distribution for Nav1.5 α‐subunits expressed alone (Figure 2) is consistent with surface‐expressed Nav 1.5 α‐subunits forming dimers, as the most common arrangement within the clusters. Significantly however, both the cluster radii and nearest‐neighbor distributions extended considerably further than these modal values. This indicates an additional layer of supramolecular organization, within the clusters in which multiple Nav channels lie close together. However, it should be noted that an accurate estimate of the absolute number of Nav1.5 α‐subunits per cluster is not straightforward as nanoscopic stoichiometry is challenging in STORM imaging. The major issues include the unspecific labeling ratio between fluorophore and the protein of interest, and over counting of proteins due to multiple blinks from single fluorophores.^34^

We suggest that on the two‐dimensional surface of the plasma membrane, the Nav1.5 α‐subunit dimers themselves further interact. It is becoming clear that such weak homophilic interactions are common for plasma membrane proteins.^35^ Such homophilic interactions could explain the fact that several mutations in the Nav1.5 α‐subunit that underlie inherited cardiopathologies, act in a strongly dominant‐negative capacity, in which the mutant α‐subunit binds to and interferes directly with the trafficking and/or gating behavior of the wild‐type channel. Significantly, this occurs even when the mutant and wild‐type Nav1.5 channels are co‐expressed in HEK293F cells.^28^, ^30^ The data presented here indicate that clustering may be an inherent property of Nav1.5 channels.

We have previously suggested that the presence of multiple β3‐subunits could act to enhance local Nav1.5 α‐subunit oligomerization on the plasma membrane.^3^, ^10^ However, our STORM data indicate that the β3‐subunit did not affect the relative proportion of Nav1.5 α‐subunits in clusters, nor the relative density of clusters on the plasma membrane (Figure 2). Nevertheless, the β3‐subunit did affect cluster structure. First, it significantly increased the distribution of cluster radii, shifting the modal peak of the distribution from 5 to 10 nm to a higher peak‐radii of 10‐15 nm. Second, it significantly altered the form of the distribution of nearest‐neighbor distances between α‐subunits within clusters, generating a bimodal distribution with distinct maximum values of ~6 and ~12 nm, respectively (Figure 2E). The persistence of the 6 nm peak could suggest that the Nav1.5 α‐subunits retain their dimeric association. However, the additional 12 nm modal peak in the nearest‐neighbor distribution suggests an additional alteration in the relative arrangement of α‐subunits within the clusters, perhaps indicating that β3 is now promoting particular orientations of Nav1.5 α‐subunit dimers, within existing clusters.

The presence of β3 generated an enhanced PLA signal between Nav1.5 α‐subunits compared to that in the absence of β3 (Figure 3A,B). Since our STORM data indicated that β3 did not increase the proportion of oligomerized Nav1.5 α‐subunits or cluster density (Figure 2B,C), this suggests that the enhanced PLA signal is not the result of non‐specific “crowding” caused by over‐expression of β3‐subunits. By its nature, PLA is a highly sensitive method for detecting protein proximity.^18^ However, because PLA is based on a DNA amplification protocol, it is inevitably sensitive to even minor differences in factors such as epitope accessibility, not just for the two different labeling antibodies but also the single‐stranded DNA linker and amplification enzymes. Consequently, PLA signals compared under different conditions where the relevant epitopes adopt different relative orientations may not provide rigorously quantitative indications of proximity and should therefore be interpreted with caution.^36^ For example, there is a strongly enhanced PLA signal recorded for EGF receptors when they bind EGF ligand. This has been interpreted to indicate that EGF promotes the dimerization of the EGF receptor.^30^ However, recent STORM imaging has revealed that even in the absence of EGF, the EGF receptors form complex oligomers on the plasma membrane, but the EGF binding induced a local geometric rearrangement of these receptors such that the intracellular tyrosine kinase domains could more readily cross‐phosphorylate each other.^37^ Hence, the enhanced PLA signal following EGF binding most likely reflects this local rearrangement in the pre‐existing EGF‐receptor clusters, rather than ligand‐induced receptor dimerization. It should therefore be emphasized that in studying membrane‐bound protein assemblies, super‐resolution imaging and PLA‐type experiments potentially reflect differing aspects of these supramolecular interactions. Furthermore, as detected by standard immunofluorescence, HA‐tagged and GFP‐tagged Nav1.5 subunits co‐localize, both at the plasma membrane and within internal membrane compartments, when they are co‐expressed in HEK293F cells (Supplementary Figure S1). However, the PLA signal is consistently stronger at the plasma membrane (Figure 3), providing further evidence that the PLA signal intensity reflects not just local Nav channel proximity, but is also sensitive to additional factors such as the relative change in epitope accessibility caused by the influence in the β3‐subunit within a specific cellular compartment.

The location of the β3‐interaction sites on the Nav channel α‐subunit is currently not well defined. However, we have previously demonstrated that the β3‐subunit can potentially bind to the Nav1.5 α‐subunit at one or more sites on the transmembrane domain and/or the intracellular region of β3.^13^ In the case of Nav1.1, the β3 intracellular region binds to the α‐subunit C‐terminal domain,^38^ which lies directly underneath the DIV voltage sensor.^39^ The β3 intracellular region is 32 amino acids long and is largely unstructured.^5^ Therefore, if the β3 intracellular region similarly binds to the Nav1.5 C‐terminal domain, then one of the β3‐binding sites on the α‐subunit must lie within a radius of no more than about 7‐8 nm from the α‐subunit C‐terminal domain. The voltage sensors from DI, DIII, and DIV all lie comfortably within this distance.^39^

Further insights have also recently been provided from cryo‐EM structures of the eel and human Nav1.4 channel α‐subunits and their associations with a single β1‐subunit.^33^, ^40^ Here, the transmembrane domain of the β1‐subunit interacts with the S1 and S2 transmembrane helices of the DIII voltage sensor and the Ig domain forms salt‐bridges with a short extracellular loop between these two helices. This α‐subunit binding site on the β1 Ig domain includes a region of the N‐terminus that shows sequence conservation with the trimer interface of β3. Therefore, if a β3‐subunit Ig domain were to bind to the α‐subunit at this location—and in the same manner as β1—then it would not be able to form a trimer with other β3‐subunits. However, AFM images indicate that under conditions of high expression, the β3‐subunit can bind at up to four separate sites symmetrically arranged around the Nav1.5 α‐subunit,^10^ a finding that is consistent with recent electrophysiological work that indicated more than one binding site for β3 on the Nav1.5 α‐subunit.^41^ The presence of multiple β‐subunits around the α‐subunit is likely to increase the effective “footprint” of a Nav1.5 complex and could provide a simple explanation for the increased radii distribution shown in Figure 2D.

We found that β3‐subunits had no significant effect on the *V*_½_ of steady‐state activation (Figure 4 and Table 1) but showed clear and significant depolarizing effects on the *V*_½_ of steady‐state inactivation (Figure 5 and Table 1). In contrast, the slope factors (*k*) of both steady‐state activation and inactivation remain unchanged. The slope factor term is classically attributed to the effective charge transfer (*z*) involved in transitions between resting and activated, and activated and inactivated Nav channels through the equation: *k* = RT/*z*F (R is the Gas constant; T is the absolute temperature, and F is the Faraday constant).^42^ The values of *k* for both activation and inactivation corresponded to charge transfers of *z* between 3 and 4 and were accordingly in agreement with values obtained from other studies and in other systems.^2^, ^43^ These findings are consistent with such charged groups on individual α‐subunits, likely those underlying respective voltage sensors, remaining sufficiently functionally far apart within the clusters, so as not to be altered by the presence of β3.

**Table 1:** Nav1.5 channel gating alone or with β3 co‐expressed. Parameters are derived from the Boltzmann function fit to steady‐state activation and inactivation data, and from a mono‐exponential function for recovery from inactivation data

	Activation			Inactivation			Recovery from inactivation		
	*V*_½_ (mV)	*k* (mV)	n	*V*_½_ (mV)	*k* (mV)	n	τ (ms)	*t*_½_ (ms)	n
Nav1.5 + EGFP	−42.62 ± 1.81	7.28 ± 0.61	8	−96.14 ± 0.72	−7.17 ± 0.20	27	7.64 ± 1.32	5.29 ± 0.92	7
Nav1.5 + β3‐EGFP	−42.61 ± 1.67	9.42 ± 0.94	10	−90.64 ± 1.02***	6.47 ± 0.32	20	4.28 ± 1.19***	2.97 ± 0.82***	10

Note

Data are means ± SEM, statistically significant results were determined using unpaired *t* tests.

*** *P* < .001 vs Nav1.5 + EGFP.

A similarly selective electrophysiological effect of β3 on Nav1.5 inactivation has been reported when studied in *Xenopus* oocytes.^4^, ^41^ It is also consistent with data from the Scn3b^−/−^ mouse model that lacks β3 expression and shows distinctive conduction abnormalities.^7^, ^8^ Thus, the electrophysiological effects described here mimic key features of the channel behavior in a whole animal model. The DIV voltage sensor is known to play a major role in the modulation of the voltage dependence of inactivation.^1^ Our data suggest that at least one of the electrophysiologically important β3‐binding sites will lie close to this region. As noted above, a β3 molecule tethered to the α‐subunit by its intracellular region will lie close to the DIV voltage sensor where it could affect voltage dependence of inactivation.

Such functional independence between clustered Nav1.5 α‐subunits extend to the kinetic properties of Hodgkin‐Huxley modeling, in which β3 co‐expression failed to exert significant effects on the time constants for activation and inactivation. This suggests that the geometric rearrangement of the Nav1.5 channels driven by the β3‐subunit did not change any functional coupling between α‐subunits (Figures 6 and 7). The β3‐subunit did significantly accelerate recovery from inactivation (Figure 8). However, the α‐subunit C‐terminal domain binds to both the α‐subunit inactivation gate^44^ and to the intracellular region of β3.^38^ Hence, the recovery from inactivation data could be more simply explained by interactions between a single α‐ and a single associated β3‐subunit, rather than cooperative interactions between different α‐subunits.

If Nav1.5 channels clustered together independently of β3, then any role for β3 must reflect other features of Nav channel biology. For example, the depolarizing shift in *V*_½_ inactivation caused by β3 implies that the electric field detected by the inactivation sensor has increased, the physiological consequence of which will be to increase the number of channels that remain activatable at a given voltage. If under these circumstances, the Nav1.5 α‐subunits are locally clustered, it will increase the local density of channels on a given patch of membrane and therefore increase the maximum density of sodium current capable of being delivered through that local patch. It is also possible that organizing Nav channels into clustered units will reduce the rate of channel endocytosis, and further enhance local channel density.

In conclusion, our data indicate that under conditions typical of many published electrophysiological studies, Nav1.5 channels exist as multimers both in the presence and absence of the β3‐subunit. It will be interesting to see if this phenomenon holds for other cell systems. For example, in cellular expression systems such as CHO and COS cells, β3 produces hyperpolarizing shifts in *V*_½_ of both activation and inactivation for Nav1.5.^6^, ^9^, ^13^ The reasons for this opposite and strikingly different behavior compared to HEK293F cells, oocytes and native cardiomyocytes are completely unknown, but it could involve differences in post‐translational modifications and/or accessory proteins. But it could also involve different degrees of heterogenetity in Nav channel oligomerization. At a time when near atomic‐resolution structures for Nav channels are beginning to be solved, we emphasize that the supramolecular organization of the channels on the cellular plasma membrane should also be considered.

## AUTHOR CONTRIBUTIONS

S.C. Salvage, A.J. Thompson, C.L.H. Huang, and A.P. Jackson designed research; S.C. Salvage, A. McStea, and J.R. Irons performed the experimental work; M. Hirsch, L. Wang, C. Tynan, and M. Martin‐Fernandez analyzed and interpreted the super‐resolution imaging data; M.W. Reed and R. Butler developed software for data analysis; S.C. Salvage, J.S. Rees, A.J. Thompson, C.L.H. Huang, and A.P. Jackson interpreted the data; S.C. Salvage, C.L.H. Huang, and A.P. Jackson wrote the paper.

## CONFLICT OF INTEREST

The authors declare no competing interests.
