# Supplementary figures and images for: Supramolecular clustering of the cardiac sodium channel Nav1.5 in HEK293F cells, with and without the auxiliary β3‐subunit
Source: FASEB J. 2020 Jan 16;34(3):3537–53. doi: 10.1096/fj.201701473RR (PMC7079131; doi:10.1096/fj.201701473RR)

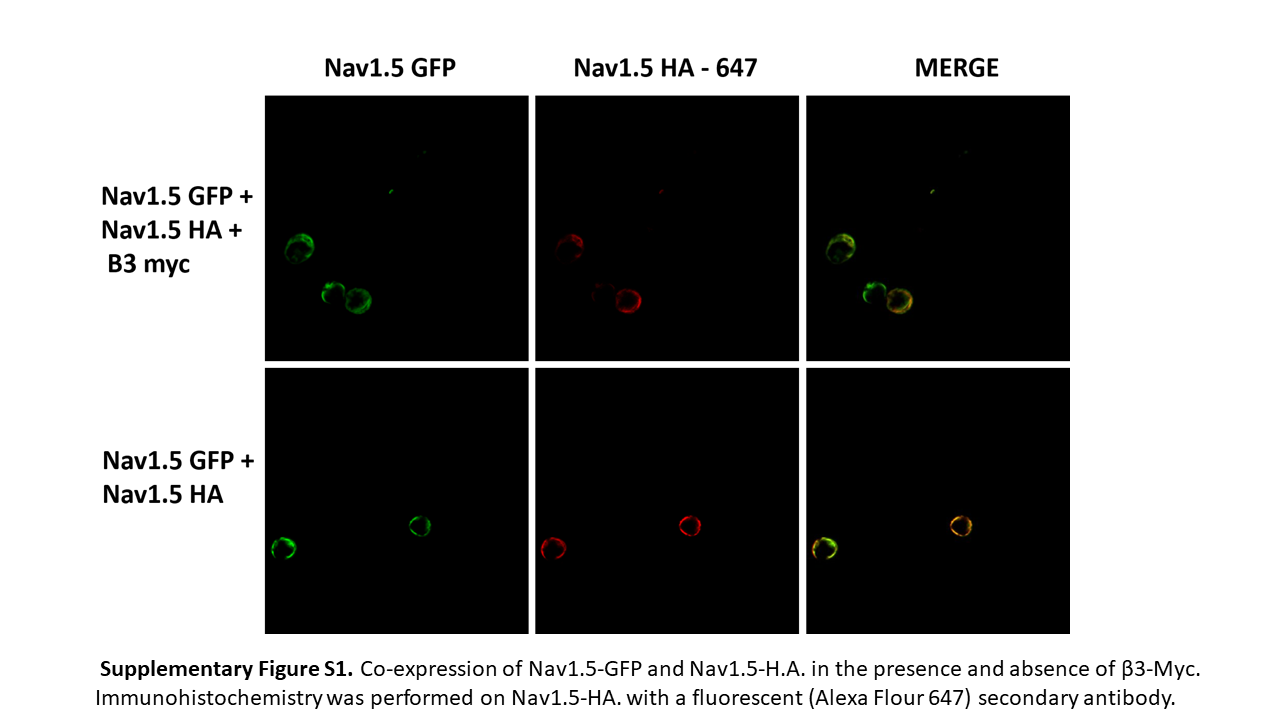

Supplement: Supplementary file 1 [file FSB2-34-3537-s001.png]
